# Integrating HIV, hepatitis B and syphilis screening and treatment through the Maternal, Newborn and Child Health platform to reach global elimination targets

**DOI:** 10.5365/wpsar.2017.8.3.005

**Published:** 2017-12-31

**Authors:** Joseph Woodring, Naoko Ishikawa, Mari Nagai, Maya Malarski, Yoshihiro Takashima, Howard Sobel, Ying-Ru Lo

**Affiliations:** a Division of Communicable Disease, World Health Organization Regional Office for the Western Pacific, Manila, Philippines.; b Division of Building Healthy Communities and Populations, World Health Organization Regional Office for the Western Pacific, Manila, Philippines.; c Imperial College London, London, England.

## Abstract

Every year, an estimated 180 000 babies in the Western Pacific Region are infected by hepatitis B, 13 000 by syphilis and 1400 by HIV through mother-to-child transmission. (*1*) These infections can be largely prevented by antenatal screening, treatment and timely vaccination for newborns. Despite challenges in controlling each disease, major achievements have been made. National immunization programmes have reduced the regional hepatitis B prevalence from over 8% in 1990 to 0.93% among children born in 2012. In addition, HIV testing and treatment have helped keep the regional prevalence of HIV infections at 0.1%. In contrast, the number of maternal syphilis cases is still high in the Western Pacific Region, with an estimated 45 million cases in 2012. Elimination of mother-to-child transmission of these infections cannot be achieved through vertically applied programming and require using and augmenting to the shared Maternal, Newborn and Child Health platform to coordinate, integrate and enable cost efficiencies for these elimination efforts. The *Regional Framework for Triple Elimination of Mother-to-Child Transmission of HIV, Hepatitis B and Syphilis in Asia and the Pacific 2018–2030* offers such a coordinated approach towards achieving the triple elimination of mother-to-child transmission of HIV, hepatitis B and syphilis and provides guidance for decision-makers, managers and health professionals working in programmes addressing maternal, newborn and child health, HIV, hepatitis, sexually transmitted infections and immunization.

The *Regional Framework for Triple Elimination of Mother-to-Child Transmission of HIV, Hepatitis B and Syphilis in Asia and the Pacific 2018–2030* (*2*) (Triple Elimination Framework) was endorsed by all Member States at the sixty-eighth session of the Regional Committee for the Western Pacific. It was developed to provide a coordinated approach to achieve and sustain elimination of these largely preventable infections using the shared Maternal, Newborn and Child Health (MNCH) platform for planning, service delivery, monitoring and evaluation. With nearly nine out of 10 mothers and children in this Region already receiving antenatal, perinatal, postnatal and well-baby care services, it is more efficient to build additional prevention services upon the shared platform than delivering them as single uncoordinated interventions solely through traditional, vertical, disease-specific control and surveillance programmes.

Endorsed by the World Health Assembly in 2016, the 2030 elimination targets for the *Global Health Sector Strategy on HIV 2016–2021*, the *Global Health Sector Strategy on Viral Hepatitis 2016–2021* and the *Global Health Sector Strategy on Sexually Transmitted Infections 2016–2021* include: 0.1% or lower hepatitis B surface antigen (HBsAg) prevalence among children and 50 or fewer cases per 100 000 live births for paediatric HIV infections and congenital syphilis. (*3*-*5*)

These three diseases have a significant burden in the Western Pacific Region: the Region alone accounts for 45% of all global hepatitis B infections; (*2*) an increasing trend of syphilis infections is observed among key populations including women of reproductive age; (*6*) and while HIV prevalence is low throughout the Region at 0.1%, the HIV mother-to-child transmission (MTCT) rate is high at 12%. (*7*)

MNCH care has made significant progress in the Region. From 1990 through 2015, the maternal mortality ratio decreased by 64% from 114 to 41 maternal deaths per 100 000 live births, (*8*) in part due to the increases in antenatal care coverage and births attended by skilled birth attendants. Nearly nine in 10 pregnant women in the Region have attended at least one antenatal care visit and have delivered in a health facility, while provision of quality services and access to at least four antenatal visits still remain as challenges. (*9*) DTP3 vaccine coverage for children has remained over 95% since 2009 with 97.3% coverage in 2016. (*10*) These multiple entry points in receiving peripartum services provide a unique opportunity for coordination and integration of HIV, hepatitis B and syphilis interventions to move towards elimination of mother-to-child transmission (EMTCT) of these infections.

The Region has shown remarkable progress with national immunization programmes, reducing the regional HBsAg prevalence to less than 1% among children born in 2012. Not all countries met the 2012 or 2017 regional prevalence targets among 5-year-olds (less than 2% and less than 1%, respectively) or the regional 2017 milestones of 95% or higher hepatitis B birth dose and 95% or higher hepatitis B third-dose vaccine coverage. (*11*, *12*) Thirty countries had evidence of meeting the 2012 goal of less than 2%; as of November 2017, 18 countries have been verified as meeting the 2017 goal of less than 1%, with five additional countries having evidence of meeting this same goal. Introduction of additional interventions are likely to be required to reach the 0.1% HBsAg prevalence elimination target by 2030, including antenatal HBsAg screening, antiviral treatment of pregnant women with high viral loads and the use of hepatitis B immunoglobulin among infants born to HBsAg-positive mothers. (*4*) Modelling has shown that global elimination of hepatitis B as a major public health threat can only be achieved by scaling up hepatitis B vaccine third-dose coverage to 90% and birth-dose coverage to 80%, peripartum antivirals to 80% of hepatitis B e-antigen-positive mothers and increasing testing and treatment to 80% of those eligible. (*13*) To meet these suggested screening and treatment targets, immunizations programmes must work with MNCH and sexually transmitted infection programmes through an integrated effort to reach hepatitis B EMTCT.

In 2014, WHO established the global criteria for dual EMTCT of HIV and syphilis that were further updated in 2017. (*14*) Several countries were already validated as having achieved elimination. In this Region, EMTCT of HIV and syphilis has seen limited progress to date. With the target of a 90% reduction in new HIV infections among infants by 2015, actual reductions have only been 27%. (*2*) Maternal and congenital infections decreased by one-third from 2008 to 2012; however, coverage of antenatal syphilis screening and treatment remains low in several countries in the Region. (*15*)

Antenatal HIV and syphilis screening coverage and hepatitis B birth-dose coverage were assessed between October 2016 and June 2017 in 161 randomly selected health facilities that had introduced Early Essential Newborn Care (EENC) in Cambodia, China, the Lao People's Democratic Republic, Mongolia, Papua New Guinea, the Philippines, Solomon Islands and Viet Nam. Accounting for 97% of all neonatal deaths in the Region, these eight countries have been selected as priority countries since 2014 under the *Action Plan for Healthy Newborn Infants in the Western Pacific Region (2014–2020)*. (*16*) Hepatitis B birth-dose vaccination has been promoted through EENC coaching to health workers dealing with intrapartum and postnatal care. (*17*) **Fig. 1** shows that hepatitis B birth-dose coverage was higher than syphilis and HIV antenatal screening coverage in seven of eight countries, with China having 100% coverage for all three. This shows that coordination among the different programmes can improve access to essential services for both women and their babies, while lack of collaboration could result in limited access and inefficiencies.

**Fig. 1:**
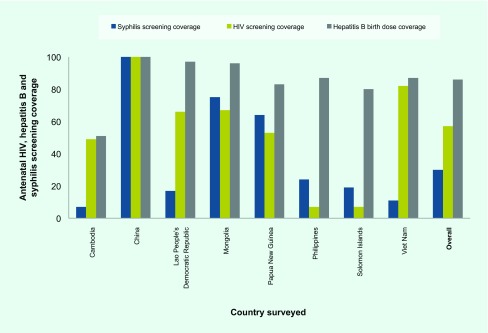
Antenatal HIV and syphilis screening coverage and hepatitis B birth-dose coverage in eight countries in the Western Pacific Region

Some countries in the Region have already begun pioneering a coordinated approach to triple elimination. For example, China has an EMTCT strategy that integrates provision of the essential package of services for universal HIV, hepatitis B and syphilis screening where all three tests are offered concurrently and free of charge. Further interventions such as HIV and syphilis treatment, including hepatitis B prophylaxis and follow-up testing and care for mothers and their children are provided for free. As a result, MTCT of HIV decreased to 6.7% in 2013, and over 1200 paediatric HIV infections were averted in 2014. (*18*) Mongolia has developed national guidelines for HIV, syphilis and hepatitis B and C antenatal screening, recommending antiviral treatment of women with high viral loads and hepatitis B immunoglobulin to infants born to these mothers. These underpin the importance of coordination and collaboration among concerned programmes for better health outcomes for mother and child. (*1*)

Current interventions must be scaled up substantially, other interventions introduced and coordination among programmes improved to achieve the global EMTCT targets. (*1*, *13*) In response, the Triple Elimination Framework proposes a vision to provide every child the greatest chance to start a healthy life free of three preventable communicable diseases. By better coordinating service delivery among programmes and including the incorporation of hepatitis B screening into existing HIV and syphilis screening at antenatal clinic, the Triple Elimination Framework looks to integrate these programmes to enable pregnant women to know their own and their partners’ infection status. It also allows pregnant mothers to understand and receive the necessary interventions for themselves and their baby during pregnancy, delivery and postnatally and to ensure that their babies receive these necessary interventions to prevent transmission of these infections (**Fig. 2**).

**Fig. 2:**
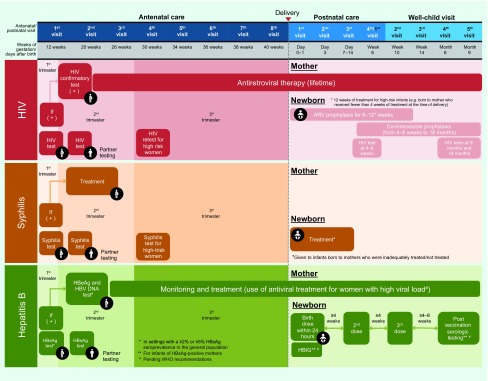
HIV, syphilis and hepatitis B screening, treatment and vaccination services offered during antenatal, delivery, postnatal care and well child visits

The Triple Elimination Framework suggests a set of key indicators under the headings of policy, impact and programme for monitoring and evaluating EMTCT. This includes eventually developing global guidance that incorporates hepatitis B into WHO established criteria for EMTCT of HIV and syphilis. The Triple Elimination Framework will also need to be supplemented by an economic analysis of the introduction of additional interventions for EMTCT of hepatitis B. This is particularly pertinent for countries with high hepatitis B vaccine birth-dose and third-dose coverage rates that are looking to expand their perinatal programmes.

Moving towards triple elimination should result in greater collaboration between programmes and thus improve accessibility, effectiveness, efficiency and sustainability of MNCH services for every woman, child and their family.

## Ethics statement

The findings and conclusions in this report are those of the authors and do not necessarily represent the views of the World Health Organization (WHO) or the WHO regional offices.

## References

[1] Provisional agenda item 12. Triple elimination of mother-to-child transmission of HIV, hepatitis B and syphilis. In: Sixty-eighth session of the WHO Regional Committee for the Western Pacific, Brisbane, Australia 9–13 October 2017. Manila: WHO Regional Office for the Western Pacific; 2017 (http://www.wpro.who.int/about/regional_committee/68/documents/wpr_rc68_7_hiv_hepa_syphilis.pdf, accessed 20 December 2017).

[2] Regional framework for the triple elimination of mother-to-child transmission of HIV, hepatitis B and syphilis in Asia and the Pacific 2018–2030 [DRAFT]. Manila: WHO Regional Office for the Western Pacific; 2017 (http://www.wpro.who.int/about/regional_committee/68/documents/wpr_rc68_7_annex_hiv_hepa_syphilis.pdf?ua=1, accessed 9 September 2017).

[3] Global health sector strategy on HIV, 2016–2021. Geneva: World Health Organization; 2016 (http://apps.who.int/iris/bitstream/10665/246178/1/WHO-HIV-2016.05-eng.pdf, accessed 9 September 2017).

[4] Global health sector strategy on viral hepatitis, 2016–2021. Geneva: World Health Organization; 2016 (http://apps.who.int/iris/bitstream/10665/246177/1/WHO-HIV-2016.06-eng.pdf?ua=1, accessed 9 September 2017).

[5] Global health sector strategy on sexually transmitted infections, 2016–2021. Geneva: World Health Organization; 2016 (http://apps.who.int/iris/bitstream/10665/246296/1/WHO-RHR-16.09-eng.pdf, accessed 9 September 2017).

[6] Wijesooriya NS, Rochat RW, Kamb ML, Turlapati P, Temmerman M, Broutet N, et al. Global burden of maternal and congenital syphilis in 2008 and 2012: a health systems modelling study. Lancet Glob Health. 2016 8;4(8):e525–33. 10.1016/S2214-109X(16)30135-8 27443780 PMC6759483

[7] Global AIDS monitoring. Geneva: UNAIDS; 2017 (http://www.unaids.org/sites/default/files/media_asset/2017-Global-AIDS-Monitoring_en.pdf, accessed 9 September 2017).

[8] Trends in maternal mortality: 1990 to 2015: estimates by WHO, UNICEF, UNFPA, World Bank Group and the United Nations Population Division. Geneva: World Health Organization; 2015 (http://www.who.int/reproductivehealth/publications/monitoring/maternal-mortality-2015/en/, accessed 1 November 2017).

[9] UNICEF data: monitoring the situation of children and women. New York: UNICEF; 2017 (http://data.unicef.org/topic/maternal-health/antenatal-care, accessed 31 May 2017).

[10] WHO/UNICEF joint reporting form on immunization. Geneva: World Health Organization and New York, NY: UNICEF; 2017 (http://apps.who.int/immunization_monitoring/globalsummary/timeseries/tscoveragedtp3.html, accessed 31 May 2017).

[11] Wiesen E, Diorditsa S, Li X. Progress towards hepatitis B prevention through vaccination in the Western Pacific, 1990-2014. Vaccine. 2016 5 27;34(25):2855–62. 10.1016/j.vaccine.2016.03.060 27020710

[12] Regional action plan for viral hepatitis in the Western Pacific 2016–2020. Manila: WHO Regional Office for the Western Pacific; 2016 (http://www.wpro.who.int/hepatitis/resource/features/regional_action_plan/en/, accessed 4 September 2017).

[13] Nayagam S, Thursz M, Sicuri E, Conteh L, Wiktor S, Low-Beer D, et al. Requirements for global elimination of hepatitis B: a modelling study. Lancet Infect Dis. 2016 12;16(12):1399–408. 10.1016/S1473-3099(16)30204-3 27638356

[14] Global guidance on criteria and processes for validation. Elimination of mother-to-child transmission of HIV and syphilis, second edition. Geneva: World Health Organization; 2017 (http://www.who.int/reproductivehealth/publications/emtct-hiv-syphilis/en/, accessed 11 December 2017).

[15] UNICEF. WHO, UNFPA and UNAIDS. Progress review and roadmap: elimination of parent-to-child transmission of HIV and syphilis in Asia and the Pacific in 2015 and beyond. Bangkok: UNICEF East Asia and Pacific Regional Office; 2016 (http://www.wpro.who.int/hiv/documents/topics/pmtct/20160920-eptct-progress-report/en/)

[16] Action plan for healthy newborn infants in the Western Pacific Region (2014–2020). Manila: WHO Regional Office for the Western Pacific; 2014 (http://www.wpro.who.int/child_adolescent_health/documents/regional_action_plan_newborn/en/, accessed 1 November 2017).

[17] Early essential newborn care: clinical practice pocket guide. Manila: WHO Regional Office for the Western Pacific; 2014 (http://iris.wpro.who.int/handle/10665.1/10798, accessed 1 November 2017).

[18] Wang AL, Qiao YP, Wang LH, Fang LW, Wang F, Jin X, et al. Integrated prevention of mother-to-child transmission for human immunodeficiency virus, syphilis and hepatitis B virus in China. Bull World Health Organ. 2015 1 01;93(1):52–6. 10.2471/BLT.14.139626 25558108 PMC4271682

